# A Practical Anodic and Cathodic Curve Intersection Model to Understand Multiple Corrosion Potentials of Fe-Based Glassy Alloys in OH^-^ Contained Solutions

**DOI:** 10.1371/journal.pone.0146421

**Published:** 2016-01-15

**Authors:** Y. J. Li, Y. G. Wang, B. An, H. Xu, Y. Liu, L. C. Zhang, H. Y. Ma, W. M. Wang

**Affiliations:** 1 Key Laboratory for Liquid-Solid Structural Evolution and Processing of Materials, Ministry of Education, Shandong University, Jinan 250061, China; 2 School of Engineering, Edith Cowan University, 270 Joondalup Drive, Joondalup, Perth, WA6027, Australia; 3 School of Chemistry and Chemical Engineering, Shandong University, Jinan, Shandong 250100, China; The University of Akron, UNITED STATES

## Abstract

A practical anodic and cathodic curve intersection model, which consisted of an apparent anodic curve and an imaginary cathodic line, was proposed to explain multiple corrosion potentials occurred in potentiodynamic polarization curves of Fe-based glassy alloys in alkaline solution. The apparent anodic curve was selected from the measured anodic curves. The imaginary cathodic line was obtained by linearly fitting the differences of anodic curves and can be moved evenly or rotated to predict the number and value of corrosion potentials.

## Introduction

Fe-based glassy alloys have been widely used in industry because of their high glass-forming ability, soft magnetic properties, good corrosion and wear resistance and ultrahigh strength properties [1–5]. Recently, the Fe_78_Si_9_B_13_ metallic glass is the leading product in Chinese Fe-based amorphous ribbons market [6]. In 1988 year, Yoshizawa and co-workers [1] invent the alloy labeled as Finemet with the composition of Fe_73.5_Si_13.5_B_9_Cu_1_Nb_3_, which has a super soft magnetic property and is used widely in electronic devices. For Fe-based glassy alloys, there exhibits a clear passive zone in the polarization curve in OH^-^ contained solutions, while the passive zone is very short in the solution without OH^-^ [5]. Meanwhile, the corrosion resistance becomes an important factor to consider in using Fe-based glasses [7,8]. Hence, it is valuable to study the electrochemical behavior of Fe_78_Si_9_B_13_ and Fe_73.5_Si_13.5_B_9_Cu_1_Nb_3_ glasses in different environments.

Measuring potentiodynamic polarization curve is one of the traditional methods to evaluate electrochemical properties [9,10]. And many works on multiple corrosion potentials in polarization curve for stainless steels in acidic solution have been reported in the literature [11–15]. Regarding the influencing factors on the corrosion potentials, Escrivà-Cerdán et al. [16] have found that high-alloyed austenitic stainless steel UNS N08031 (Alloy 31) has three corrosion potentials in phosphoric acid at 60 and 80°C since the temperature can favor the cathodic reaction. Qiao et al. [17] have reported that a certain sulfuric acid and oxygen concentration can induce three corrosion potentials during the polarization of a nitrogen bearing stainless steel. It has been found that the corrosion behavior of pure titanium shows three corrosion potentials when corroded in the H_2_SO_4_ solution containing 0.001 M and 0.002 M fluride ions [18]. In addition, the speed jet of phosphoric acid solution to the stainless steel can also induce three corrosion potentials [19]. However, to the authors’ knowledge, three corrosion potentials occurring in the alkaline solution for Fe-based glassy alloys have not been reported so far.

The occurrence of multiple corrosion potentials was reported to be attributed to the instability of passive film. Kelly et al. [20] have reported that the formation mechanism of three corrosion potentials is that the anodic and cathodic Evans lines intersect at three points and there exist one anodic loop and one cathodic loop between the three corrosion potentials. It was also pointed that the origin of the cathodic loop is related to the greater rate of the cathodic reaction than that of the passive current density and the former can conceal the latter at these potentials near the active-passive transition. Qiao et al. [17] have constructed ideal polarization curve models based on the aforementioned mechanism to understand the appearance of three corrosion potentials under certain sulfuric acid concentration and oxygen content. However, the authors have not given the direct relation between the ideal polarization curve and experimental data, which is valuable to discover.

In addition, many works have pointed out that the scanning rate and solution concentration have a significant impact on electrochemical behavior. Zhang et al. [21] have found that the scanning rate can directly affect the Tafel slope. It have been confirmed by Manning et al. that the pitting potential and scanning rate have a functional relationship [22]. In addition, Nakagawa et al. [23] have pointed that the fluoride concentration and pH can affect the polarization test of titanium in NaF solution with various concentrations and pH values.

In this study, our major aim is to build a practical accurate model to explain the occurrence of multiple corrosion potentials of Fe-based glassy ribbons when scanning rate (*ν*) or NaOH concentration (*c*_NaOH_) reaches a specific value. In our model, we process a new method to construct the imaginary cathodic line from the measured anodic polarization curves.

## Materials and Methods

The commercial Fe_78_Si_9_B_13_ glassy ribbons and master alloys were supplied by Qingdao Yunlu Energy Technology Company Ltd. The original size of the commercial ribbons was 40 mm in width, 35 μm in thickness and about 20000 mm in length, from which the ribbon samples with size of 3×40 mm were cut for electrochemical tests. Fe_78_Si_9_B_13_ master alloy was prepared by melting iron, pure silicon and Fe-B alloy in medium induction furnace, and Fe_73.5_Si_13.5_B_9_Cu_1_Nb_3_ master alloy was obtained by melting of pure iron, silicon, copper, niobium and ferroboron alloy. The Fe_73.5_Si_13.5_B_9_Cu_1_Nb_3_ glassy ribbons were obtained using a single copper roller in the lab. X-ray diffraction analysis (as shown in Refs [24] and [25]) proves that the as–spun ribbon samples are in fully amorphous state. Since the most of metallic materials in the industrial application are in crystalline state. In this paper, we focus on the metallic glasses, which are sparser than the metallic crystalline materials; by the way, we are also interested in the electrochemical behavior of their crystalline counterpart, i.e. master alloys. Hence, we choose the master alloys as the comparison.

All electrochemical measurements were carried out using a standard three–electrode system: working electrode, platinum counter electrode and Hg/HgO reference electrode. All the measurements were only conducted on the wheel sides of the ribbons and their free sides were covered by silicone rubber. CHI 660E electrochemical workstation was used for measuring the polarization curves. All experiments were performed at room temperature (298 K). In the measurement of potentiodynamic polarization curves with a series of scanning rates (*ν*), the electrolyte was 0.6 M NaCl + 0.12 M NaOH. In the measurement of polarization curves with different NaOH concentrations (*c*_NaOH_), *ν* was fixed at 1 mV/s and the electrolyte was 0.6 M NaCl + *x* M NaOH (0.04 ≤ *x* ≤ 0.7). All measurements were repeated at least three times to ensure good reproducibility.

To understand the anodic process of Fe_78_Si_9_B_13_ glassy ribbon in 0.6 M NaCl + 0.12 M NaOH, the ribbons were polarized to three different potentials which were located after the first anodic peak, 2ad anodic peak and three corrosion potentials, respectively. The sample surface morphology was examined using scanning electron microscopy (SEM, SU-70). The surface was also analyzed by X-ray photoelectron spectroscopy (XPS, ESCALAB 250Xi) performed on a photoelectron spectrometer with Al-Kα excitation.

## Results

### Polarization with different scanning rates

**Fig 1:**
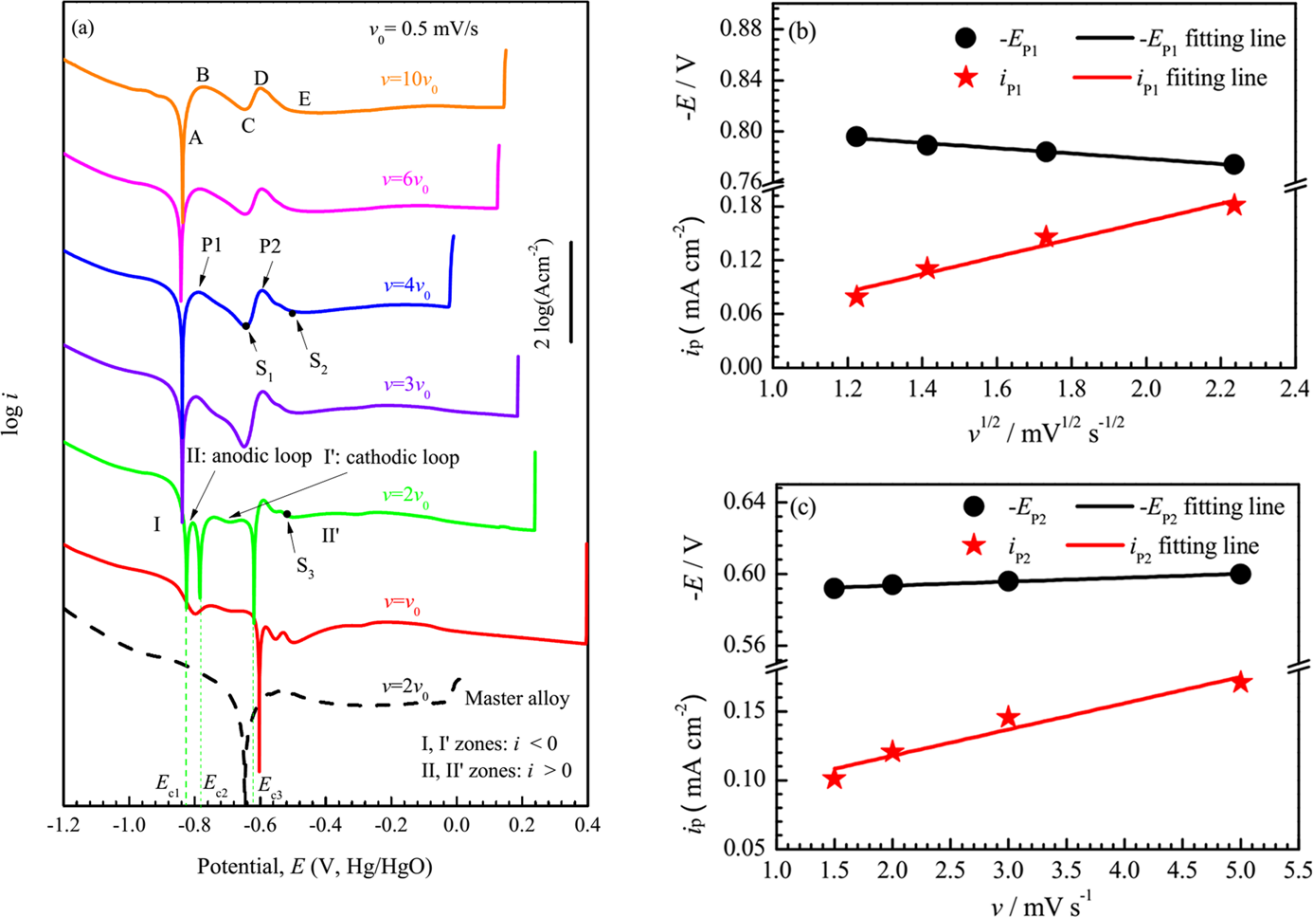
Electrochemical tests of samples. (a) Potentiodynamic polarization curves of Fe_78_Si_9_B_13_ glassy ribbons and the corresponding master alloy with different scanning rates *ν* in 0.6 M NaCl + 0.12 M NaOH solution. For clarity, the curves of Fe_78_Si_9_B_13_ glassy ribbons with *ν* = *ν*_0_, 2 *ν*_0_, 3 *ν*_0_, 4 *ν*_0_, 6 *ν*_0_ and 10 *ν*_0_ are shifted upward by multiplying the raw data with 10^2^, 10^4^, 10^6^, 10^8^, 10^10^ and 10^12^ respectively. (b) Variation of the current densities *i*_P1_ and peak potentials *E*_P1_ with the square root of *ν*. (c) Variation of the current densities *i*_P2_ and peak potentials *E*_P2_ with *ν*. P1 and P2 indicate the two current density peaks such as A-B-C and C-D-E zones in the polarization curve with *ν* = 10 *ν*_0_ (*ν*_0_ = 0.5 mV/s), respectively.

Fig 1A shows the electrochemical polarization curves of Fe_78_Si_9_B_13_ glassy ribbons in 0.6 M NaCl + 0.12 M NaOH solution with the scanning rates *ν* = 0.5–5 mV/s. As *ν* is reduced from 5 to 1.5 mV/s, the polarization curves have the similar shapes with two current density peaks P1 and P2 in A-B-C and C-D-E zones, respectively. As *ν* was further reduced to 1 mV/s, three corrosion potentials appear with the sacrifice of P1, and the polarization curve is divided into I, II, I’ and II’ zones. The current density in both I and I’ zones is negative, and that in II and II’ is positive. Hence, II is called anodic loop and I’ is called cathodic loop. The curve with *ν* = 0.5 mV/s has only one corrosion potential, and there exist one and two small troughs before and after it respectively. It should be noted that, the measured polarization curve of Fe_78_Si_9_B_13_ master alloy with *ν* = 1 mV/s has one corrosion potential, and no distinct current density peak P1 or P2 (Fig 1A). This indicates that the occurrence of three corrosion potentials is not only related to *ν*, but also to microstructure.

Moreover, in Fig 1A, as the scanning rate *v* is decreased from 5 to 2 mV/s, the pitting potential *E*_pit_ decreases, whereas *E*_pit_ increases with further decreasing *v* from 1.5 to 0.05 mV/s. In the first changing range of *v*, the positive relationship between *E*_pit_ and *v* can be explained by the point defect medol (PDM) [26,27]. However, in the second range, it is found that the passive current density *i*_pass_ is apparently lower than that in the first range, and according to the argument that cumulative anodic change density, *Q*_c_, is constant, which is presented as [28]_:_

$$Q_{c}\approx i_{\mathrm{pass}}\times \Delta t_{p}=i_{\mathrm{pass}}\frac{(E_{\mathrm{pt}}-E_{\mathrm{ocp}})\times 10^{-3}{}^{}}{v}$$ (1)

where *E*_ocp_ is the open circuit potential, Δ*t*_p_ is the anodic polarization time duration until the stable pitting. Now we get:

$$E_{\mathrm{pit}}=\frac{Q_{c}\cdot v}{i_{\mathrm{pass}}}+E_{\mathrm{ocp}}$$ (2)

When *i*_pass_ has a larger decreasing rate than the decreasing rate of *v*, *E*_pit_ might increase. Hence, it is understood that *E*_pit_ increases when *v* varies from 1.5–0.05 mV/s. This phenomenon is similar to the fact that *Q*_c_ and *E*_pit_ sometimes increase abnormally with decreasing *v* in the experimental data of Zhang and co-workers’ work [26].

As seen from Fig 1B, the values of current density *i*_p_ and the corresponding potential *E*_p_ of P1 vary linearly with the square root of the scanning rate *ν*. According to Müller and Calandra’s model [29,30], P1 can be explained by ohmic resistance caused by the insoluble reaction products on the sample surface and the *i*_p1_ and *E*_p1_ can be expressed as

ip1=(nFρkM)12(1−θp)ν12 (3)

Ep1=(nFρkM)12[(δk)+R0A0(1−θp)]ν12 (4)

where *k* is the specific conductivity of the solution within the pores of the film, *ρ* the film density, *δ* the film thickness, *M* the molar mass of the film formed by the flow of Coulombs, *R*_0_ the external resistance, *θ*_P_ the surface fractional coverage and *A*_0_ the electrode area.

Fig 1C shows that the *i*_p_ of P2 decreases linearly but the corresponding *E*_p_ decreases slightly with the decrease of the scanning rate. According to Srinivasan and Gileadi’s model [31], P2 is related to an adsorption process and the *i*_p2_ and *E*_p2_ can be defined by

$$i_{\text{p}2}=\left(\frac{\gamma F}{4RT}\right)\nu$$ (5)

$$E_{\text{p}2}=-\left(\frac{RT}{F}\right)\text{In}K$$ (6)

where *K* is the velocity constant ratio of adsorption to desorption, *γ* is the charge required to form a monolayer of adsorbed species and other symbols have their usual meaning in electrochemistry.

**Fig 2:**
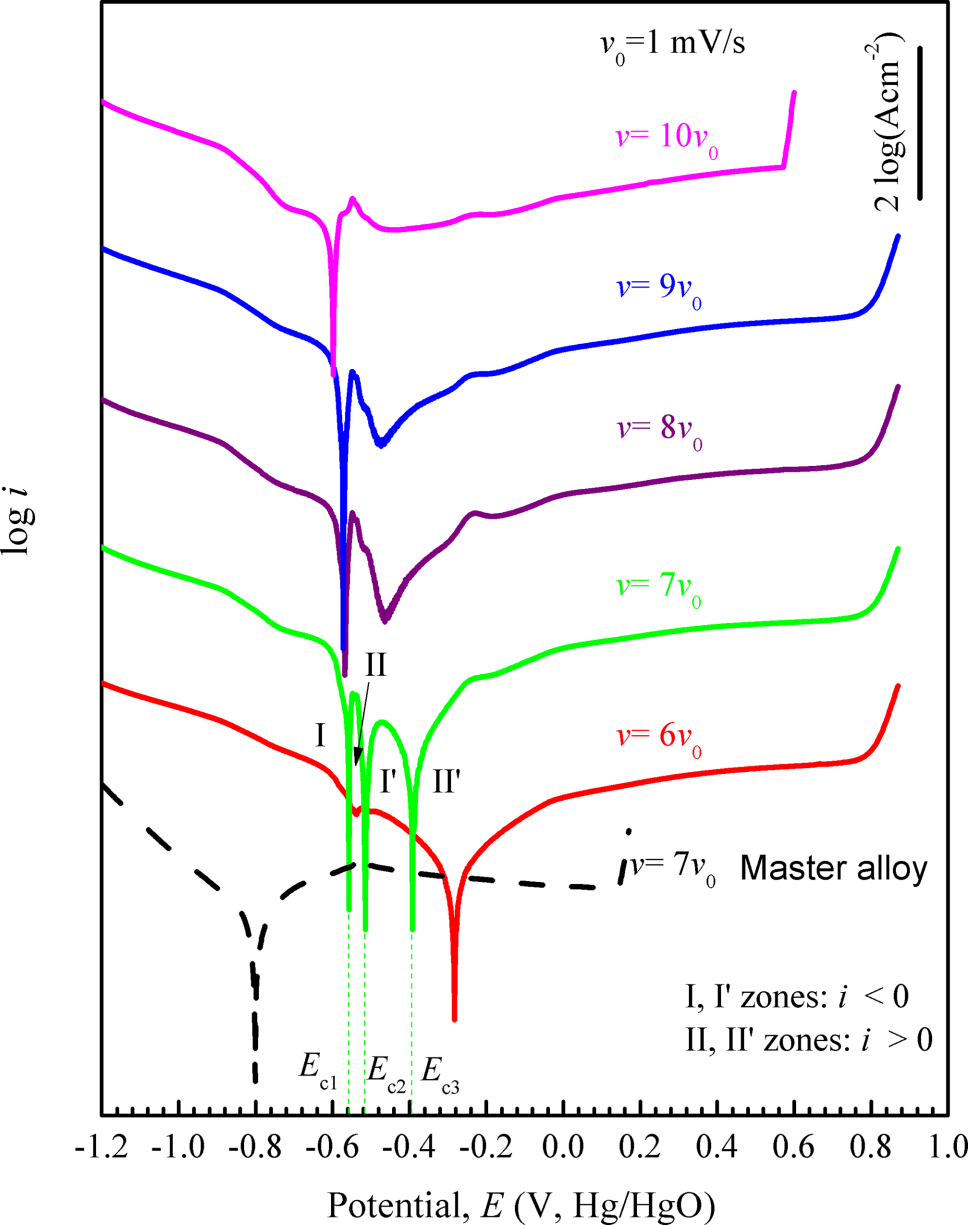
Potentiodynamic polarization curves of Fe_73.5_Si_13.5_B_9_Cu_1_Nb_3_ glassy ribbons and the corresponding master alloy with different scanning rates *ν* in 0.6 M NaCl + 0.12 M NaOH solution. For clarity, the curves of Fe_73.5_Si_13.5_B_9_Cu_1_Nb_3_ glassy ribbons with *ν* = 6 *ν*_0_, 7 *ν*_0_, 8 *ν*_0_, 9 *ν*_0_ and 10 *ν*_0_ are shifted upward by multiplying the raw data with 10^2^, 10^4^, 10^6^, 10^8^ and 10^10^ respectively.

In addition, the polarization curves of Fe_73.5_Si_13.5_B_9_Cu_1_Nb_3_ glassy ribbons in 0.6 M NaCl + 0.12 M NaOH solution with *ν* = 6–10 mV/s are shown in Fig 2. The polarization curves with *ν* = 8–10 mV/s have a similar shape with one current density peak. With decreasing *ν*, the current density of the peak decreases, but its potential approximately remains constant. As *ν* decreases to 7 mV/s, three corrosion potentials present like Fe_78_Si_9_B_13_ glassy ribbon with 1 mV/s and they divide the polarization curve into I, II, I’ and II’ zones with the current density symbol of negative, positive, negative and positive, respectively. The polarization curve with *ν* = 6 mV/s has only one corrosion potential with a small trough before it. Similarly, the polarization curve of Fe_73.5_Si_13.5_B_9_Cu_1_Nb_3_ master alloy with 7 mV/s does not have three corrosion potentials or a distinct current density peak in anodic part. In addition, the *E*_pit_ of samples creases with decreasing *v*, which is similar to the phenomenon in the second changing range of *v* in Fe_78_Si_9_B_13_ (Fig 1A).

### Polarization with different NaOH concentrations

**Fig 3:**
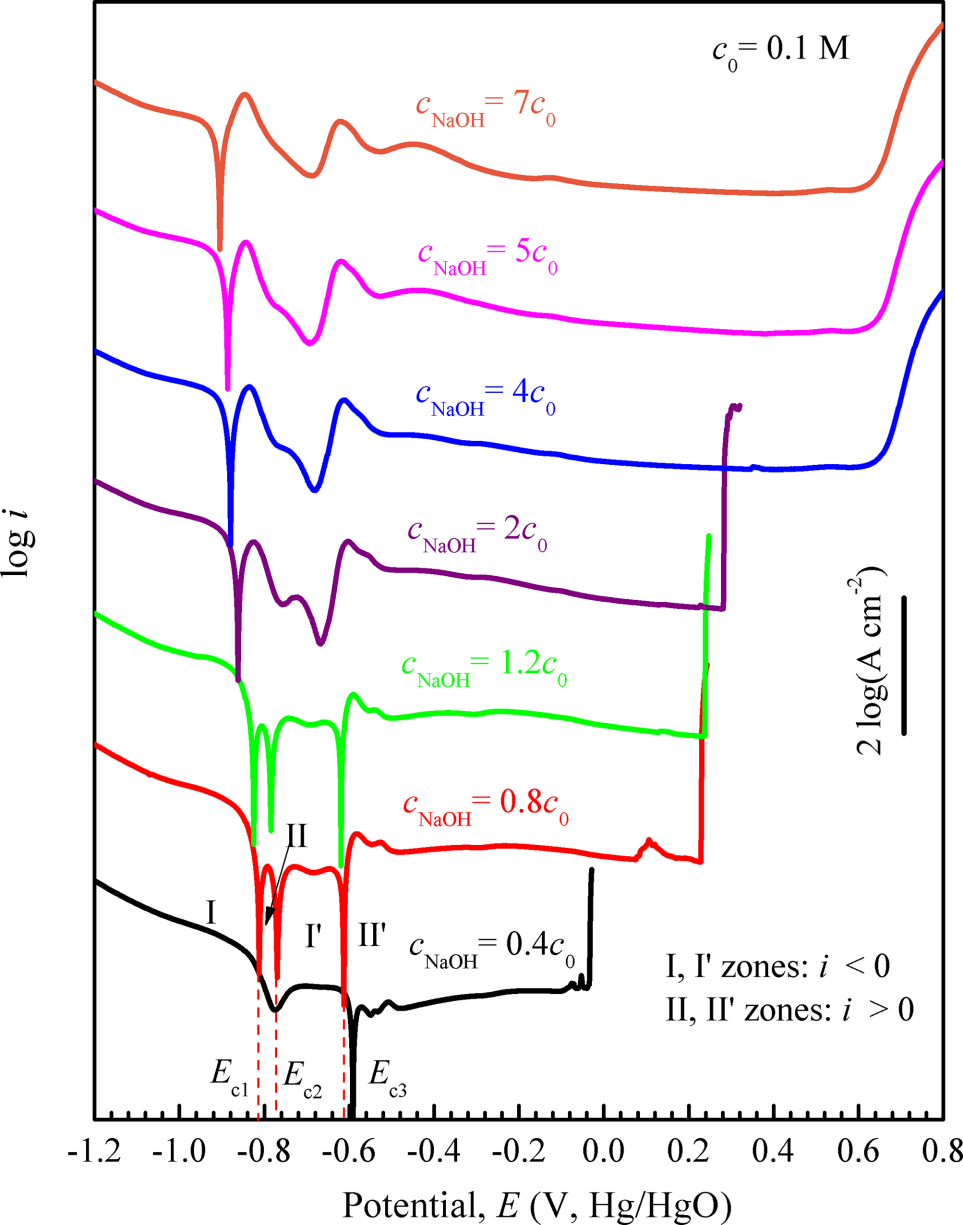
Potentiodynamic polarization curves of Fe_78_Si_9_B_13_ glassy ribbons in 0.6 M NaCl + *x* M NaOH (*x* = 0.04–0.7) solution with *ν* = 1mV/s. For clarity, the curves with *c* = 0.8 *c*_0_, 1.2 *c*_0_, 2 *c*_0_, 4 *c*_0_, 5 *c*_0_ and 7 *c*_0_ are shifted upward by multiplying the raw data with 10^2^, 10^4^, 10^6^, 10^8^, 10^10^ and 10^12^ respectively.

Fig 3 displays the polarization curves of Fe_78_Si_9_B_13_ glassy alloys in 0.6 M NaCl + *x* M NaOH solutions with the scanning rate *ν* = 1 mV/s. The characteristic of the polarization curve in Fig 3 as well as those in Figs 1A and 2 are listed in Table 1. The curves with *c*_NaOH_ = 0.2–0.7 M have two current density peaks as the same as those obtained in 0.6 M NaCl + 0.12 M NaOH solution with *ν* = 5 to 1.5 mV/s. With decreasing *c*_NaOH_, the corrosion potential and both two peaks’ potentials shift to right, and the passive range shortens. When *c*_NaOH_ decreases to 0.2 M, hydrolysis reaction near 0.6 V transforms into pitting corrosion reaction. Three corrosion potentials *E*_c1_, *E*_c2_ and *E*_c3_ occur and divide the polarization curve into I, II, I’ and II’ zones looped with negative and positive symbols when *c*_NaOH_ decreases to 0.12 and 0.08 M. Apparently, the values of *E*_c1_, *E*_c2_ or *E*_c3_ with *c*_NaOH_ = 0.08 M respectively are higher than the corresponding one with *c*_NaOH_ = 0.12 M. When *c*_NaOH_ decreases to 0.04 M, the number of potentials decreases to one and the pitting potential *E*_pit_ becomes further negative. The OH^-^ dependent *E*_pit_ obeys the following relation between the pitting potential and the PH of the electrolyte [32]:

$$E_{\text{pit}}=a+b\text{PH}$$ (7)

where the value of *a* is 690 mV and *b* is 21 mV for Sn_66_Ni_34_ alloy in NaCl solution with various pH values. We use Eq (7) to give only a qualitative explanation on the *c*_NaOH_ dependent *E*_pit_ is present paper.

**Table 1:** Variation of polarization parameters of Fe_78_Si_9_B_13_ and Fe_73.5_Si_13.5_B_9_Cu_1_Nb_3_ glassy ribbons.

Alloy	*v* or *c*_NaOH_	*E*_p1_ (Vvs.Hg/HgO)	log*i*_p1_(A cm^-2^)	*E*_p2_ (Vvs.Hg/HgO)	log*i*_p2_(A cm^-2^)	log*i*_pass_(A cm^-2^)	*E*_pit_(Vvs.Hg/HgO)
Fe_78_Si_9_B_13_	10*v*_0_	-0.77±0.01	-3.74±0.11	-0.60±0.01	-3.77±0.10	-4.13±0.10	0.14±0.03
	6*v*_0_	-0.78±0.01	-3.84±0.15	-0.59±0.02	-3.84±0.30	-4.17±0.05	0.13±0.04
	4*v*_0_	-0.78±0.02	-3.96±0.12	-0.59±0.01	-3.92±0.14	-4.21±0.12	-0.02±0.03
	3*v*_0_	-0.79±0.01	-4.20±0.06	-0.59±0.01	-4.00±0.01	-4.31±0.04	0.19±0.05
	2*v*_0_	-	-	-0.59±0.01	-4.02±0.2	-4.52±0.03	0.24±0.03
	*v*_0_	-	-	-0.58±0.00	-4.83±0.3	-5.72±0.02	0.40±0.01
Fe_73.5_Si_13.5_B_9_Cu_1_Nb_3_	10*v*’_0_	-0.55±0.01	-4.67±0.01	-	-	-4.29±0.01	0.57±0.01
	9*v*’_0_	-0.55±0.01	-4.99±0.01	-	-	-4.38±0.01	0.79±0.01
	8*v*’_0_	-0.55±0.02	-4.80±0.01	-	-	-4.38±0.01	0.78±0.02
	7*v*’_0_	-	-	-	-	-4.35±0.05	0.79±0.01
	6*v*’_0_	-	-	-	-	-4.36±0.01	0.80±0.01
Fe_78_Si_9_B_13_	7*c*_0_	-0.84±0.01	-3.38±0.01	-0.62±0.02	-3.78±0.06	-4.68±0.01	0.60±0.01
	5*c*_0_	-0.84±0.01	-3.58±0.01	-0.62±0.02	-3.88±0.01	-4.75±0.01	0.62±0.01
	4*c*_0_	-0.83±0.01	-3.71±0.01	-0.61±0.02	-3.89±0.05	-4.79±0.01	0.64±0.01
	2*c*_0_	-0.83±0.01	-4.12±0.06	-0.60±0.01	-4.05±0.05	-4.82±0.01	0.28±0.01
	1.2*c*_0_	-	-	-0.58±0.01	-4.60±0.07	-4.69±0.01	0.24±0.03
	0.8*c*_0_	-	-	-0.59±0.01	-4.22±0.04	-4.59±0.01	0.23±0.01
	0.4*c*_0_	-	-	-0.57±0.01	-4.47±0.01	-4.55±0.01	-0.03±0.01

*v*_0_ = 0.5 mV/s

*v*’_0_ = 1 mV/s

*c*_0_ = 0.1 M NaOH.

For clarity, the characteric parameters of the measured polarization curves of Fe_78_Si_9_B_13_ and Fe_73.5_Si_13.5_B_9_Cu_1_Nb_3_ glassy samples with various scanning rates and *c*_NaOH_ as well as their standard deviation are listed in Table 1.

### SEM and XPS measurement after various polarizations

**Fig 4:**
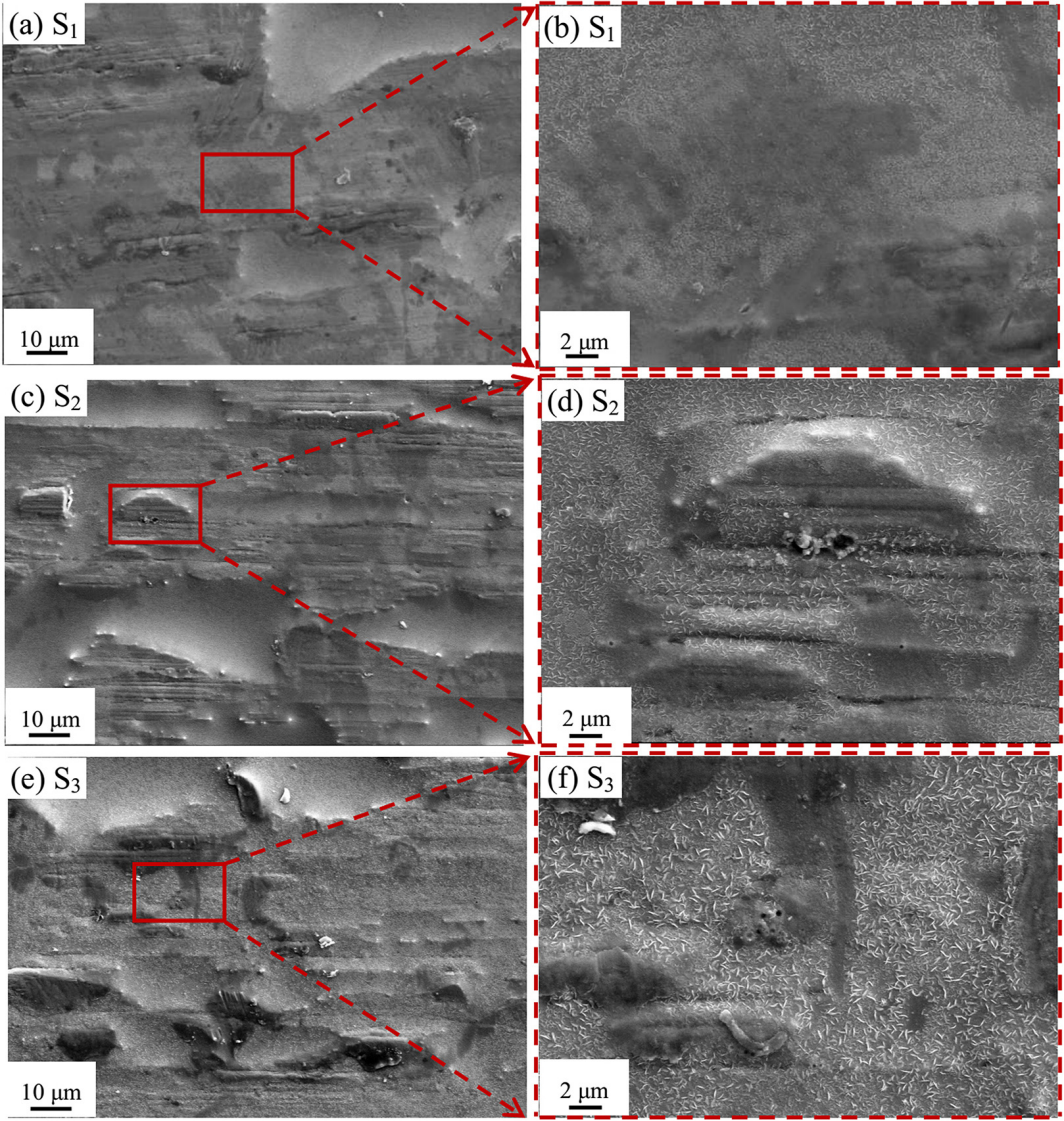
SEM micrographs for the surfaces of (a) and (b) S_1_, (c) and (d) S_2_, and (e) and (f) S_3_ samples. S_1_ and S_2_ were polarized with *ν* = 2 mV/s until the end of the first and second anodic peaks, respectively, while S_3_ was polarized to a similar potential to S_2_ with *ν* = 1 mV/s after experiencing three corrosion potentials. S_1_, S_2_ and S_3_ are indicated in Fig 1A.

In order to investigate the nature of the anodic current density peaks of Fe_78_Si_9_B_13_ glassy alloys in 0.6 M NaCl + 0.12 M NaOH, the scanning rate *ν* = 1 mV/s and 2 mV/s were chosen to polarize the glassy ribbons until three different points S_1_, S_2_ and S_3_, which are indicated in Fig 1A. Samples S_1_ and S_2_ were polarized with *ν* = 2 mV/s until the end of the first and second anodic peaks, respectively; while sample S_3_ was polarized to a similar potential to S_2_ with *ν* = 1 mV/s after experiencing three corrosion potentials. The surface morphologies of these samples are shown in Fig 4. A lot of white flake-like products in size of about 0.1–1 μm present on all the samples and their size, i.e. the surface roughness of samples, increases in order of S_1_ < S_2_ < S_3_. Meanwhile, the surface morphology of S_3_ is similar to that of S_2_ rather than S_1_.

**Fig 5:**
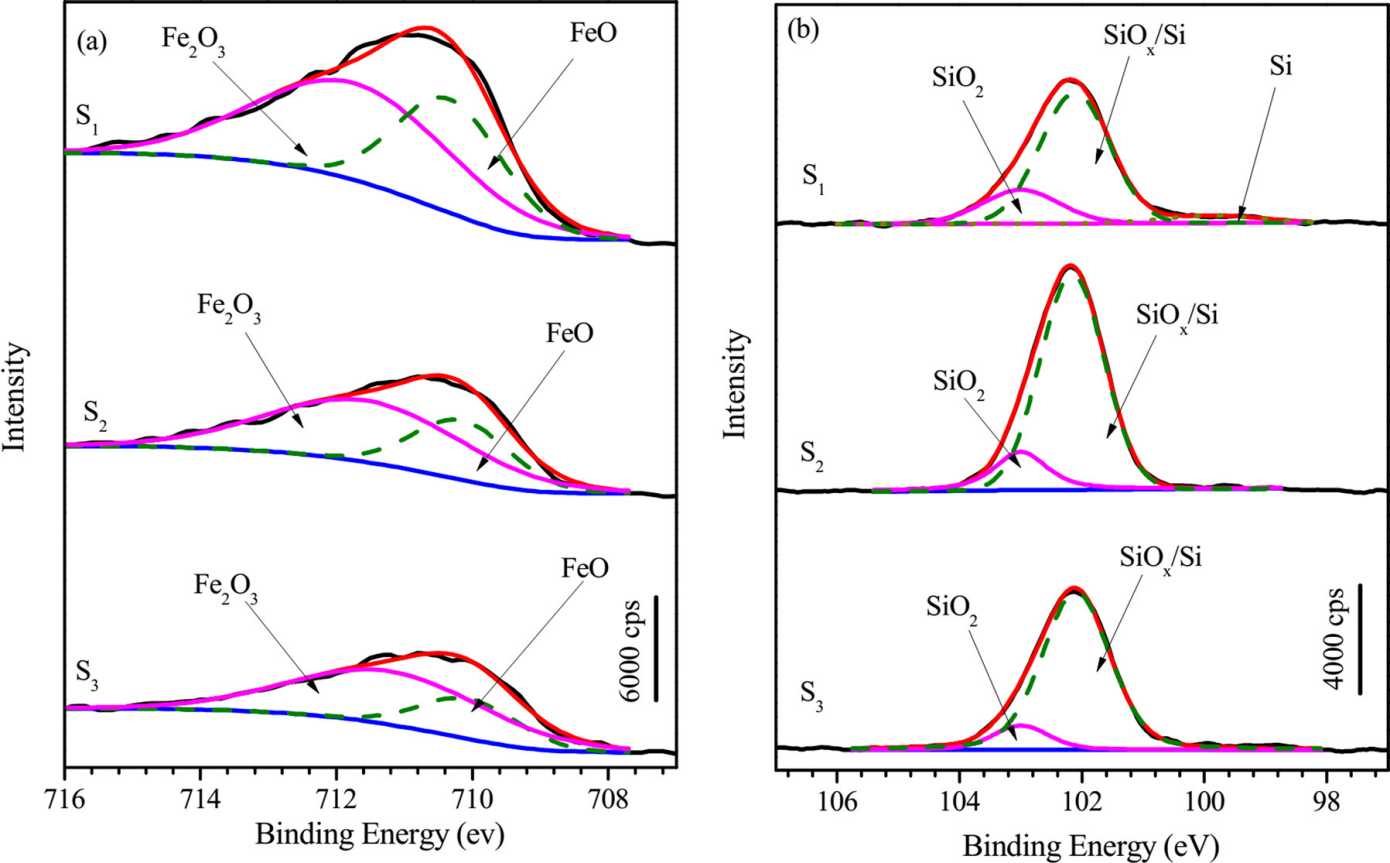
XPS spectra of (a) Fe 2p and (b) Si 2p recorded from the surfaces of S_1_, S_2_ and S_3_. S_1_ and S_2_ were polarized with *ν* = 2 mV/s until the end of the first and second anodic peaks, respectively, while S_3_ was polarized to a similar potential to S_2_ with *ν* = 1 mV/s after experiencing three corrosion potentials. S_1_, S_2_ and S_3_ are indicated in Fig 1A.

Fig 5 shows the X-ray photoelectron spectroscopy (XPS) spectra of Fe 2p and Si 2p recorded from the surfaces of S_1_, S_2_ and S_3_. For the spectra of Fe 2p, the total peak intensity of S_1_ is greater than that of S_2_ or S_3_, possibly due to the higher surface roughness of S_2_ and S_3_. The Fe 2p spectrum can be decomposed into two peaks belonging to FeO and Fe_2_O_3_ [33]. According to earlier work [34], the XPS spectrum of Si 2p of S_1_ can be decomposed into three peaks: SiO_2_, SiO_x_/Si and Si. But for S_2_ and S_3_, it can only be decomposed into two peaks corresponding to SiO_2_ and SiO_x_/Si. The decomposed peak fractions for Fe 2p and Si 2p spectra are listed in Table 2. Apparently the FeO and SiO_2_ fractions of samples change in the order S_1_ > S_2_ > S_3_, but the Fe_2_O_3_ and SiO_x_/Si fractions change oppositely. Overall, the decomposited XPS result of S_3_ is similar to that of S_2_, but different from S_1_.

**Table 2:** The fraction of decomposed peaks from XPS spectra for Fe 2p and Si 2p of the surfaces of S_1_, S_2_ and S_3_.

Sample	Fe 2p		Si 2P		
	Fe_2_O_3_ (%)	FeO (%)	SiO_2_ (%)	SiO_x_/Si (%)	Si (%)
S_1_	61.5	38.5	21.5	72.5	6
S_2_	68.2	31.8	16.2	83.8	-
S_3_	73.3	26.7	11.0	89.0	-

## Discussion

### Practical model to explain the occurrence of multiple corrosion potentials of Fe-based glassy ribbons

From Fig 1A, the polarization curves of Fe_78_Si_9_B_13_ glassy ribbons with the scanning rate *ν* ≥ 1.5 mV/s have two current density peaks P1 and P2 in the anodic part. As *ν* = 1 mV/s, there are three corrosion potentials in the polarization curve. Kelly et al. [20] have pointed that three corrosion potentials *E*_c1_, *E*_c2_, and *E*_c3_ are the intersecting points of anodic and cathodic Evans lines based on the following equation

$$i=i_{\text{a}}-i_{\text{c}}=0,\;\text{when}\;E=E_{c1},E_{c2}\;\text{and}\;E_{c3}$$ (8)

where *i*_a_ is anodic current density, *i*_c_ is cathodic current density. Similarly, Qiao et al. [17] have supposed a schematic mechanism of corrosion with three *E*_c_ by intersecting an ideal anodic polarization curve with an ideal cathodic reaction line, there are different intersection numbers by changing the former’s or latter’s position.

It’s known that the cathodic reaction in alkaline solution is the oxygen reduction reaction [35]

$${\text{O}}_{2}+2{\text{H}}_{2}\text{O}+4{\text{e}}^{\text{-}}=4{\text{OH}}^{\text{-}}$$ (9)

and /or the water reduction reaction

$$2{\text{H}}_{2}\text{O}+2{\text{e}}^{\text{-}}={\text{H}}_{2}+2\text{O}{\text{H}}^{\text{-}}$$ (10)

The cathodic part does not show the limiting value representing the oxygen reduction reaction, but shows a relatively higher current density (Fig 1A). In addition, a lot of gas bubbles can be observed on the sample corroding surface in the experiment. Both phenomena show that the cathodic reaction in alkaline solution is dominated by water reduction (reaction (10)) [35]. Hence, decreasing *c*_NaOH_ can enhance the cathodic reaction and increase the corrosion potential (Fig 3). According to Lee [36], there are three possible determining steps in reaction (10).

$${\text{H}}_{2}\text{O}+{\text{e}}^{\text{-}}={\text{H}}_{\text{ad}}+{\text{OH}}^{\text{-}}$$ (11a)

$${\text{H}}_{\text{ad}}+{\text{H}}_{2}\text{O}+{\text{e}}^{\text{-}}={\text{H}}_{2}+{\text{OH}}^{\text{-}}$$ (11b)

$$2{\text{H}}_{\text{ad}}={\text{H}}_{2}$$ (11c)

The reaction rate *U* can be written as the following formula based on reaction (11a) by neglecting the back reaction

$$U=K_{\text{f}}\;\exp\left(-n\alpha F\psi /\mathrm{RT}\right)$$ (12)

where *n* is the total number of electrons involved in the reaction, *α* the transfer coefficient, *ψ* the potential between the sample and the solution and *K*_f_ the rate constant for the forward reaction. Then the current density *i* can be written as

$$i=nFa_{{\text{H}}_{2}\text{O}}U$$ (13)

where *a*H_2_O denotes the activity of H_2_O. Combining Eqs (12) with (13), we will get

$$\log i=\log nFa_{{\text{H}}_{2}\text{O}}K_{f}+\frac{1}{2.303}RT-\frac{n\alpha F}{2.303}\psi$$ (14)

So the cathodic reaction can be represented by a line in the half logarithmic coordinates. Such linear relation in log*i*-*ψ* can be obtained in a straightforward way according to Ref. [36]. Hence, it is understood that Kelly and Qiao used the cathodic line to explain the variation of polarization curves [17,20].

However, there are several unknown parameters like *K*_f_, *α* and *a*H_2_O in present polarization process. In the following sections, we propose a simple method to deduce an imaginary cathodic line, evenly move it or rotate it, and try to discuss the evolution of the polarization curves, especially the formation of three corrosion potentials (Figs 1A, 2 and 3).

#### Evenly moving the imaginary cathodic line

As shown in Fig 1A, the active anodic peaks P1 and P2 with the scanning rate *ν* = 5 mV/s can be divided into four potential ranges, i.e., AB, BC, CD and DE. According to Lu et al. [37], the measured current density *i*(*E*) of anodic polarization curve except the steady passive ranges can be expressed by

$$i(E)=\sum_{m}i_{0-m}(E)=\sum_{m}i_{0-m}\;\exp(\frac{E-E_{0}}{B})$$ (15)

where *i*_0-m_, *E*_0_ and *B* are exchange current density, equilibrium potential and symmetry like coefficient, respectively. Generally, *B* is also denoted by

$$B=\frac{RT}{\beta F}$$ (15a)

here *β* is symmetry coefficient, *R* is gas constant, *T* is temperature, *F* is Faraday’s constant [37]. In this paper, *R*, *T* and *F* are constants, which can be move before the exponent function.

From Figs 1A and 3 and Table 1, the second current density peak varies much smaller than the first peak with the variation of *ν* and *c*_NaOH_. So P2 could be omitted in following analysis for simplification like omitting the higher terms in series expansion. Hence, the anodic polarization curves with *ν* = 5 and 3 mV/s could be described as following:

$$i=i_{0-1}e^{\beta_{1}\psi }-i_{0-2}e^{\beta_{2}\psi }$$ (16)

$$i'=i{'}_{0-1}e^{\beta {'}_{1}\psi }-i{'}_{0-2}e^{\beta {'}_{2}\psi }$$ (17)

Since the curve shape with *ν* = 5 mV/s is similar to that with *ν* = 3 mV/s, a further simplification and assumption could be made as

$$\Delta i_{0}=i_{0-1}-i{'}_{0-1}\approx i_{0-2}-i{'}_{0-2}<0$$ (18)

and

β1=β'1,β2=β'2,β2=β1+δψ (19)

hence, the current density difference Δ*i* can be expressed as

$$\Delta i=i-i'=\Delta i_{0}e^{\beta_{1}\psi }\left(1-e^{\delta }\right)$$ (20)

Herein, we set

$$\delta =e^{-a^{0}\psi }$$ (21)

where *a*^0^ is constant and *a*^0^>*β*_1_. Now, by expansing the right item in Eq (21) with Talor`s series and omitting the higher order terms, the difference Δ*i* can be expressed as

$$\Delta i=\Delta i_{0}e^{\beta_{1}\psi }\left(1-e^{e^{-a^{0}\psi }}\right)=-\Delta i_{0}e^{\beta_{1}\psi }e^{-a^{0}\psi }$$ (22)

$$\log\;\Delta i=\log\left(-\Delta i_{0}\right)+0.434\left(\beta_{1}-a^{0}\right)\psi$$ (23)

**Fig 6:**
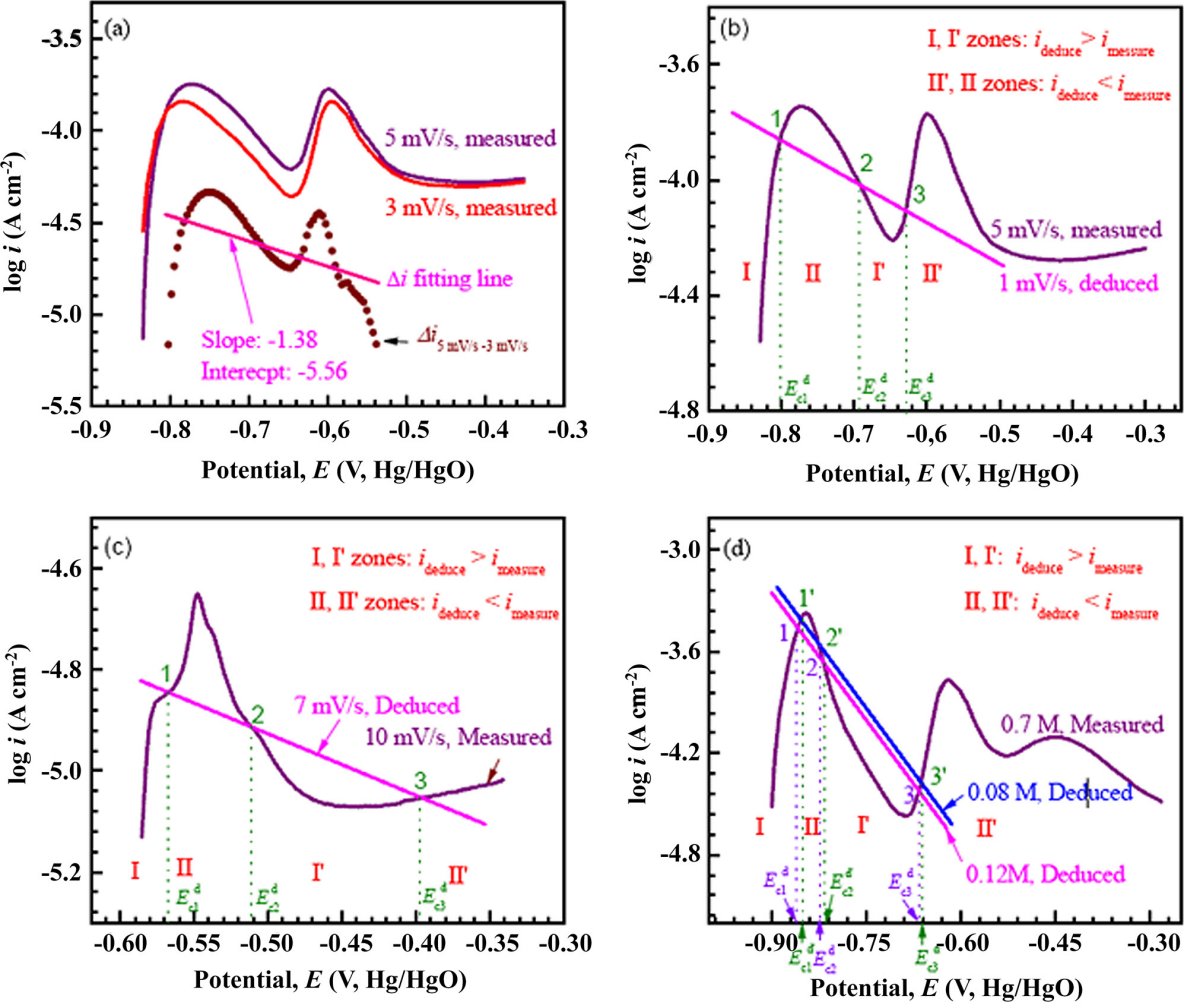
The schematic of practical cathodic and anodic curve intersection model. (a) Deducing of imaginary cathodic line from the difference of measured anodic curves; (b) deducing the corrosion potentials of Fe_78_Si_9_B_13_ glassy ribbon with 1 mV/s from that with *ν* = 5 mV/s in 0.6 M NaCl + 0.12 M NaOH solution by imposing an extrapolated imaginary cathodic line, i.e. moving evenly the imaginary cathodic line; (c) deducing the polarization curve of Fe_73.5_Si_13.5_B_9_Cu_1_Nb_3_ glassy ribbon with *ν* = 7 mV/s from that with *ν* = 10 mV/s in 0.6 M NaCl + 0.12 M NaOH solution by rotating the imaginary cathodic line; and (d) deducing the polarization curve of Fe_78_Si_9_B_13_ glassy ribbons with *c*_NaOH_ = 0.12 and 0.08 M from that with 0.7 M by rotating the imaginary cathodic line.

Eq (23) shows a liner relation between logΔ*i* and *ψ*. Its intercept and slope can be obtained by linearly fitting the difference *i*-*i*′ of the measured curves which is shown in Fig 6A. When *ν* changes, the exchange current density *i*′_0–1_ should change according to Eq (17), and then log(-Δ*i*_0_), i.e. the intercept of the Eq (23), changes; while the slope of Eq (23) does not change. In other words, the line of logΔ*i*-*ψ* moves evenly with changing *ν*.

Apparently, Eq (23) is similar to Eq (14). If Eq (20) is approximately regarded as Butler-Volmer formula, the current density difference *i* and current density *i* with *ν* = 5 mV/s can be regarded as imaginary cathodic and anodic current densities, respectively, i.e. *i*′ can be deduced if Δ*i* and *i* are known. In other words, we can predict the characteristic of a new current density *i*′ by subtracting an imaginary cathodic line *i* from the apparent cathodic curve *i* with *v* = 5 mV/s. In present work, the construction of an imaginary cathodic line is explained in the following and illustrated in Fig 6 and Table 3.

**Table 3:** The values of slope and intercept for imaginary cathodic line (ICL) for the anodic and cathodic curve intersection model of of the two samples; solution: 0.6 M NaCl+0.12 M NaOH solution.

Sample	Change of *ν*	Slope (A cm^-2^ /V)	Intercept(A cm^-2^)	Method	Category
Fe_78_Si_9_B_13_	5 mV/s → 3 mV/s	-1.37±0.18	-5.56±0.11	d.m.	
	5 mV/s → 2 mV/s	-1.33±0.10	-5.20±0.07	d.m.	
	5 mV/s → 1.5 mV/s	-1.40±0.10	-5.10±0.07	d.m.	
	5 mV/s→ 1 mV/s	-1.37	-4.95	e.d.m.	Evenly moving ICL
	5 mV/s→ 0.5 mV/s	-1.37	-4.79	e.d.m.	Evenly moving ICL
Fe_73.5_Si_13.5_B_9_ Cu_1_Nb_3_	10 mV/s → 9 mV/s	-0.87±0.18	-5.49±0.09	d.m.	
	10 mV/s → 8 mV/s	-1.05±0.03	-5.58±0.03	d.m.	
	10 mV/s → 7 mV/s	-1.23	-5.54	e.d.m.	Rotating ICL
	10 mV/s → 6 mV/s	-1.41	-5.54	e.d.m.	Rotating ICL

d.m.—from difference of measured polarization curves

e.d.m.–extrapolated from d.m.

Firstly, we subtract the curves with *ν* = 3 mV/s from that with *ν* = 5 mV/s, and fitting the difference into a line in logΔ*i*-*E* graph with a intercept and a slope as shown in Fig 6A and denoted in Table 3 with the *v* change of 5 mV/s → 3 mV/s; we also subtract the curves with *v* = 2 mV/s and 1.5 mV/s from that with *v* = 5 mV/s and obtain the groups of slopes and intercepts, which are listed in Table 3 and denoted by the *ν* changes of 5 mV/s → 2 mV/s and 5 mV/s → 1.5 mV/s, respectively. Here, the slopes fluctuate with an average value; while the intercepts decrease with decreasing *ν*, which is consistent with Eq (23). Secondly, we choose the average value of above slopes and the linearly extrapolated value from the known intercepts in Table 3 to make the imaginary cathodic line for *v* = 1 mV/s i.e. moving the known imaginary cathodic line upward evenly, which is denoted by the *ν* change of 5 mV/s → 1 mV/s.

After constructing the imaginary cathodic line for *ν* = 1 mV/s, we impose it on the apparent anodic curve with *ν* = 5 mV/s, and intersecting the apparent anodic curve at three points, and indicating that the deduced polarization curve with 1 mV/s has three corrosion potentials (Fig 6B). In addition, according to Eq (20), the current density symbol of I and I’ zones, in which the anodic curve is lower than the imaginary cathodic line, is negative, being consistent with the measured polarization curve (Fig 1A). As *ν* = 0.5 mV/s, the imaginary cathodic line, whose slope and intercept are determinded with the *ν* change of 5 mV/s → 0.5 mV/s in Table 3, intersects the apparent anodic curve at about -0.6 V_Hg/HgO_ and is nearly tangent to P1 of the apparent anodic curve at about -0.8 V_Hg/HgO_. Thus, a trough rather than*E*_c1_ or *E*_c2_ appears in the measured polarization curves with *ν* = 0.5 mV/s. In short words, the imaginary cathodic line and apparent anodic curve intersection model can be used to deduce the multiple corrosion potentials in polarization curves.

#### Rotating the imaginary cathodic line

For the polarization curves of Fe_73.5_Si_13_B_9_Cu_1_Nb_3_ glassy ribbons with various *ν* (Fig 2), the anodic and cathodic curve intersection model is also applied based on Eq (20). Here, because the curve with *ν* = 10 mV/s is similar to that with *ν* = 9 mV/s, Eqs (16) and (17) are used to describe the former and latter curves, respectively. Assuming

$$i_{0-1}=i{'}_{0-1}=\text{constant},\;i_{0-2}=i{'}_{0-2}=\text{constant}$$ (24a)

β1=β'1,β'2=β2+δ'ψ (24b)

We will get

$$\Delta i=i\text{-}i'=i_{0-2}e^{\beta_{2}\psi }\left(e^{\delta '}-1\right)$$ (25)

Here, we set

$$\delta '=e^{-a'\psi }$$ (26)

where *a*′ varies with *ν* and *a*′ >*β*_2_. Combining Eqs (25) with (26), expansing the right item of Eq (26) with Talor`s series and omitting the higher order terms, we will have

$$\log\;\Delta i=\log i_{0-2}+0.434\left(\beta_{2}-a'\right)\psi$$ (27)

Eq (27) is also similar to Eq (14), so it can be considered as an imaginary cathodic line. With changing *ν*, its intercept is constant, but its slope changes, i.e. the line rotates around the intersection with vertical axis (*ψ* = 0), which is different from Eq (23). For predicating the corrosion potential with a given *ν* (Fig 2), we use the active anodic region of the curve with 10 mV/s as the apparent anodic curve. After subtracting the measured curves with 9 and 8 mV/s and linearly fitting the differences, the intercepts and slopes of the lines in logΔ*i*-*E* can be obtained, which are listed in Table 3 and denoted by changing *ν* with 10 mV/s → 9 mV/s and 10 mV/s → 8 mV/s, respectively. Here, the variation of intercept is smaller than that of slope, which is consistent with Eq (27). So the average value of intercepts is chosen for constructing an imaginary cathodic line for *ν* = 7 mV/s, and its slope is linearly extrapolated from the first two slopes i.e. rotating the known imaginary cathodic line around its intersection with vertical axis (as shown by the line in Fig 6C and denoted by the *ν* change of 10 mV/s → 7 mV/s in Table 3). After constructing the imaginary cathodic line with *ν* = 7 mV/s, we impose it on the apparent anodic curve and get three interceptions at different potentials, which is consistent with the measured curve (Fig 2). In addition, according to Eq (25), the current density symbol of I and I’ zones is negative, being consistent with the measured polarization curve (Fig 2). As *ν* = 6 mV/s, the imaginary cathodic line, corresponding to the *ν* change of 10 mV/s → 6 mV/s in Table 3, intersects the apparent anodic curve at about -0.3 V_Hg/HgO_ and simultaneously tends to be tangent to the current density peak of the apparent anodic curve. Thus, the measured polarization curve with *ν* = 6 mV/s contains a trough before the corrosion potential.

Meanwhile, the measured corrosion potentials $E_{c1}^{m}$, $E_{c2}^{m}$ and $E_{c3}^{m}$ and the deduced ones $E_{c1}^{d}$ $E_{c2}^{d}$ and $E_{c3}^{d}$ for Fe_78_Si_9_B_13_ and Fe_73.5_Si_13.5_B_9_Cu_1_Nb_3_ glassy ribbons in 0.6 M NaCl + 0.12 M NaOH solution with *ν* = 1 and 7 mV/s are summarized in Table 4, respectively. For Fe_78_Si_9_B_13_ glassy ribbons, the deduced $E_{c1}^{d}$ or $E_{c2}^{d}$ respectively is slightly higher than the corresponding measured $E_{c1}^{m}$ or $E_{c2}^{m}$, but $E_{c3}^{d}$ is lower than $E_{c3}^{m}$. It is explained by the fact that the first peak of measured curves moves to left side with decreasing *ν* and their second peak moves to right (Fig 1A). For Fe_73.5_Si_13.5_B_9_Cu_1_Nb_3_ glassy ribbons, each measured corrosion potential is very close to the deduced counterpart, which is consistent with the fact that the current density peaks of the polarization curves approximately have no lateral displacement (Fig 2).

**Table 4:** The measured and deduced value $E_{corr}^{m}$ and $E_{corr}^{d}$ for the two samples; solution: 0.6 M NaCl + 0.12 M NaOH solution.

Sample	*v*	$E_{c1}^{m}$ (mV_Hg/HgO_)	$E_{c1}^{d}$ (mV_Hg/HgO_)	$E_{c2}^{m}$ (mV_Hg/HgO_)	$E_{c2}^{d}$ (mV_Hg/HgO_)	$E_{c3}^{m}$ (mV_Hg/HgO_)	$E_{c3}^{d}$ (mV_Hg/HgO_)
Fe_78_Si_9_B_13_	1 mV/s	-824	-802	-784	-690	-618	-630
Fe_73.5_Si_13.5_B_9_Cu_1_Nb_3_	7 mV/s	-558	-568	-514	-511	-392	-398

In addition, using the anodic and cathodic curve intersection model, the evolution of polarization curves with various hydroxyl concentrations can also be analyzed by the imaginary cathodic line with Eqs (23) or (27). The active anodic region of the curve obtained in the solution with *c*_NaOH_ = 0.7 M is chosen as the apparent anodic curve. After subtracting the curves obtained in solutions with *c*_NaOH_ = 0.5, 0.4 and 0.2 M and linearly fitting the difference, various intercepts and slopes could be obtained, which are summarized in Table 5 and denoted by the *c*_NaOH_ changes of 0.7 M → 0.5 M, 0.7 M → 0.4 M and 0.7 M → 0.2 M, respectively. The intercept decreases slightly with the decrease of NaOH concentration, which is opposite to the desired varying tendency of an imaginary cathodic line in our intersection model. Hence, to deduce the corrosion potentials with *c*_NaOH_ = 0.12, 0.08 and 0.04 M, we select Eq (27) as the starting point, set the intercept as a constant equal to the average value of the first three intercepts, and linearly extrapolate the slope by the first three slopes i.e. rotating the known imaginary cathodic line around its intersection with vertical axis (*ψ* = 0 (as shown by the lines in Fig 6D and denoted by the *c*_NaOH_ changes of 0.7 M → 0.12 M and 0.7 M → 0.08 M in Table 5)).

**Table 5:** The values of slope and intercept for imaginary cathodic line (ICL) for the anodic and cathodic curve intersection model of Fe_78_Si_9_B_13_ glassy ribbon; solution: 0.6 M NaCl + *x* M NaOH.

Change of *c*_NaOH_	Slope(A cm^-2^ /V)	Intercept(A cm^-2^)	Method	Category
0.7 M → 0.5 M	-4.18±0.15	-7.57±0.11	d.m.	
0.7 M → 0.4 M	-4.42±0.20	-7.71±0.15	d.m.	
0.7 M → 0.2 M	-4.81±0.25	-7.92±0.19	d.m.	
0.7 M → 0.12 M	-4.98	-7.73	e.d.m.	Rotating ICL
0.7 M → 0.08 M	-5.06	-7.73	e.d.m.	Rotating ICL
0.7 M → 0.04 M	-5.15	-7.73	e.d.m.	Rotating ICL

d.m.—from difference of measured polarization curves

e.d.m.–extrapolated from d.m.

After getting the imaginary cathodic line, the anodic and cathodic curve intersection model can be used to deduce the corrosion potentials with 0.12 and 0.08 M NaOH solutions (Fig 6D). As *c*_NaOH_ = 0.12 and 0.08 M, both the imaginary cathodic lines whose slope and intercept are determinded with the *c*_NaOH_ changes of 0.7 M → 0.12 M and 0.7 M → 0.08 M, respectively in Table 5, intersect the apparent anodic curve at three corrosion potentials (Fig 6D). According to Eq (25), the deduced current density symbol of I and I’ zones is negative and consistent with the measured polarization curve (Fig 3). As *c*_NaOH_ = 0.04 M, the imaginary cathodic line, whose slope and intercept are determinded with the *c*_NaOH_ change of 0.7 M → 0.04 M in Table 5, intersects the apparent anodic curve at about -0.6 V_Hg/HgO_ and is nearly to be tangent to P1 of the apparent anodic curve, being consistent with the emergence of the trough in the measured polarization curve with *c*_NaOH_ = 0.04 M. The measured and deduced corrosion potentials of 0.12 and 0.08 M are summarized in Table 6. Apparently, three corrosion potentials *E*_c1_, *E*_c2_ and *E*_c3_ in the polarization curves are successfully predicted. The deduced corrosion potential $E_{c1}^{d}$, $E_{c2}^{d}$ or $E_{c3}^{d}$ is slightly lower than the corresponding measured $E_{c1}^{m}$, $E_{c2}^{m}$ or $E_{c3}^{m}$ respectively, which is consistent with the fact that two current density peaks move to right with decreasing *c*_NaOH_ (Fig 3).

**Table 6:** The measured and deduced value $E_{corr}^{m}$ and $E_{corr}^{d}$ for Fe_78_Si_9_B_13_ glassy ribbons; solution: 0.6 M NaCl + 0.12 and 0.08 M NaOH.

*c*_NaOH_	$E_{c1}^{m}$ (mV_Hg/HgO_)	$E_{c1}^{d}$ (mV_Hg/HgO_)	$E_{c2}^{m}$ (mV_Hg/HgO_)	$E_{c2}^{d}$ (mV_Hg/HgO_)	$E_{c3}^{m}$ (mV_Hg/HgO_)	$E_{c3}^{d}$ (mV_Hg/HgO_)
0.12 M	-824	-858	-784	-819	-618	-667
0.08 M	-812	-855	-770	-825	-614	-663

It should be noted that choosing the starting polarization curve is very important in our anodic and cathodic curve intersection model. If the *ν* or *c*_NaOH_ of the starting polarization curves is far beyond the *ν* or *c*_NaOH_ for multiple potentials phenomenon, our model will become invalid. Moreover, if the *ν* or *c*_NaOH_ of the starting polarization curves is far below the *ν* or *c*_NaOH_ for the multiple potentials phenomenon, our model will fail to predict it. It should be noted that the applicable *v* range of our intersection model for Fe_78_Si_9_B_13_ ribbon is different from that for Fe_73.5_Si_13.5_B_9_Cu_1_Nb_3_ ribbon, due to the occurrence of their multiple corrosion potentials at different *v*.

### Nature of the first and second peaks in the anodic polarization curves of Fe-Si-B glassy ribbons

According to the previous explanation about the anodic dissolution of Fe-based alloys in alkaline solution [38], the first current density peak (P1) can be expressed

$$\text{Fe}+2{\text{OH}}^{\text{-}}⇌\text{Fe}{\left(\text{OH}\right)}_{2}+2{\text{e}}^{\text{-}}$$ (28)

$$3\text{Fe}+8{\text{OH}}^{\text{-}}⇌{\text{Fe}}_{2}{\text{O}}_{3}\cdot \text{FeO}+4{\text{H}}_{2}\text{O}+8{\text{e}}^{\text{-}}$$ (29)

$$\text{Fe}+2{\text{OH}}^{\text{-}}⇌\text{FeO}+{\text{H}}_{2}\text{O}+2{\text{e}}^{\text{-}}$$ (30)

$$3\text{Fe}{\left(\text{OH}\right)}_{2}+2{\text{OH}}^{\text{-}}⇌{\text{Fe}}_{2}{\text{O}}_{3}\cdot \text{FeO}+4{\text{H}}_{2}\text{O}+2\;{\text{e}}^{\text{-}}$$ (31)

Kelsall et al. [39] have reported that the oxidation of silicon in steel is in preference to iron at low potentials. In present case, Eq (32) should be included in the first current peak P1.

$$\text{Si}+2{\text{H}}_{2}\text{O}⇌{\text{SiO}}_{2}+4{\text{H}}^{+}+4{\text{e}}^{\text{-}}$$ (32)

Since SiO_2_ bears a high chemical stability and a dielectric character [40,41], it is understood that the first peak is an ohmic resistance controlled reaction (Fig 1B).

For the second peak (P2), it is a process of the formation of stable Fe_2_O_3_ [42], and can be expressed

$${\text{Fe}}_{2}{\text{O}}_{3}\cdot \text{FeO}+4{\text{OH}}^{\text{-}}⇌3{\text{FeO}}_{2}{}^{\text{-}}+2{\text{H}}_{2}\text{O}+{\text{e}}^{\text{-}}$$ (33)

$$2{\text{Fe}}_{2}{\text{O}}_{3}\cdot \text{FeO}+2{\text{OH}}^{\text{-}}⇌3{\text{Fe}}_{2}{\text{O}}_{3}+{\text{H}}_{2}\text{O}+2{\text{e}}^{\text{-}}$$ (34)

Kelsall et al. [39,43] have pointed out that Fe_2_SiO_4_ can be formed from iron oxide and SiO_2_/Si (IV) species at higher potentials. So the second current peak P2 should also contain the following reaction

$${\text{Fe}}_{2}{\text{O}}_{3}+{\text{SiO}}_{2}+2{\text{H}}^{+}+2{\text{e}}^{\text{-}}⇌{\text{Fe}}_{2}{\text{SiO}}_{4}+{\text{H}}_{2}\text{O}$$ (35)

According to the previous paper [44], both Eqs (33) and (34) are controlled by the adsorption step of OH^-^, and it is expected that the second peak is characterized as the adsorption controlled reaction (Fig 1C).

As shown in Fig 1A, sample S_1_ has only experienced the first peak (P1) with the scanning rate *ν* = 2 mV/s in 0.6 M NaCl + 0.12 M NaOH solution, S_2_ has experienced the first and second peaks (P1 and P2) with *ν* = 2 mV/s. As indicated by reactions (28)–(32), the first current peak P1 is the formation process of FeO, Fe_2_O_3_ and SiO_2_. And the second current peak P2 is the conversion process from FeO to Fe_2_O_3_ (Eqs (33) and (34)). In addition, the formation of P2 consumes some SiO_2_ according to Eq (35). These reactions are confirmed by the higher SiO_2_ and FeO fractions of S_1_ in XPS results compared with S_2_ (Fig 5 and Table 2).

Sample S_3_ has experienced three corrosion potentials (*E*_c1_, *E*_c2_ and *E*_c3_) and P2, ending the polarization at a similar potential to S_2_ with *ν* = 1 mV/s. The XPS decomposition similarity between S_2_ and S_3_ (Fig 5 and Table 2) indicates that, although the polarization curves of S_3_ contains three corrosion potentials *E*_c1_, *E*_c2_ and *E*_c3_ at the expense of the formation of P1, the anodic reaction of P1 still happened, which is hidden by the enhanced cathodic reaction. In other words, it is reasonable to obtain the measured anodic curves with *ν* = 5 mV/s as the apparent anodic curve in our practical model.

According to the above analysis, the distinct peak P1 with formation of FeO, Fe_2_O_3_ and SiO_2_ is necessary condition for the appearance of three corrosion potentials according to the practical model; while the second peak is not the necessary condition for their appearance in polarization curves. It should be noted that the formation of the anodic peak P1 or P2 is closely associated with the composition and microstructure of Fe-based alloys.

## Conclusions

Two anodic peaks (P1 and P2) present in the polarization curve of Fe_78_Si_9_B_13_ glassy ribbon in NaCl + NaOH solution when the scanning rate *ν* or NaOH concentration *c*_NaOH_ is high enough. The former peak formation is ohmic resistance controlled, which is associated with the formation of SiO_2_, and the latter is adsorption controlled, accompanying the damage of the SiO_2_ film. The formation of P1 or P2 is closely associated with the composition and microstructure of Fe-based alloys. Three corrosion potentials (*E*_c1_, *E*_c2_ and *E*_c3_) are observed in the polarization curve for Fe-based glassy ribbons in OH^-^ contained solutions when *ν* or *c*_NaOH_ reaches a specific value.

The occurrence of multiple corrosion potentials is explained by a practical anodic and cathodic curve intersection model. In this model, we choose the measured anodic curve as the apparent anodic curve, from it subtract the measured anodic curves with various *ν* or *c*_NaOH_ and linearly fit the difference as the imaginary cathodic line. By moving the cathodic line evenly or rotating it and imposing it with the apparent anodic curve, the number of intersections can be obtained and the number of corrosion potentials can be predicted. The practical model shows that the distinct P1 is the necessary condition for the occurrence of three corrosion potentials.
